# Supplementary material for: Regulation and Function of C-Type Natriuretic Peptide (CNP) in Gonadotrope-Derived Cell Lines
Source: Cells. 2019 Sep 14;8(9):1086. doi: 10.3390/cells8091086 (PMC6769446; doi:10.3390/cells8091086)
Supplement: Supplementary file 1 [file cells-08-01086-s001.pdf]

Gene	Forward primer (5'-3'), reverse primer (3'-5')	Product Size (bp)
Nppa	AGGTGACACTATAGAATACGGTACCGAAGATAACAGCC<br>GTACGACTCACTATAGGGACAGAGTGGGAGAGGCAAGAC	256
Nppb	AGGTGACACTATAGAATACACCCAAAAAGAGTCCTTCG<br>GTACGACTCACTATAGGGAAAGAGACCCAGGCAGAGTCA	249
Nppc	AGGTGACACTATAGAATAAATACAAAGGCGGCAACAAG<br>GTACGACTCACTATAGGGACGTTGGAGGTGTTTCCAGAT	221
Npr1	AGGTGACACTATAGAATACTTGGAATTCCTGAAGCAGC<br>GTACGACTCACTATAGGGACTGGACATAGAGCAGGAGCC	158
Npr2	AGGTGACACTATAGAATACCTTGATGTCTTTGGGGAGA<br>GTACGACTCACTATAGGGAGATTTGGGGGTTCTCGGTAT	186
Npr3	AGGTGACACTATAGAATATCTGCTGTCCTCTGTCCCTT<br>GTACGACTCACTATAGGGACTGGTTTTGAAGGGCATCAT	235
Furin	AGGTGACACTATAGAATAGGCTTTCATGACAACCCATT<br>GTACGACTCACTATAGGGAGGTCAGCGTCCCATAGTTGT	137
Corin	AGGTGACACTATAGAATAGAATCTTTCCATTCCGCAAA<br>GTACGACTCACTATAGGGATATCAATGAGGCAAATGGCA	263
β-Actin	AGGTGACACTATAGAATAGTACCACCATGTACCCAGGC<br>GTACGACTCACTATAGGGAGTACTTGCGCTCAGGAGGAG	144
cFos	AGGTGACACTATAGAATACTGTCCGGTTCCTTCTATGC<br>GTACGACTCACTATAGGGAAGTACAGGTGACCACGGGAG	151
cJun	AGGTGACACTATAGAATATAACAGTGGGTGCCAACTCA<br>GTACGACTCACTATAGGGATGTCGCAACCAGTCAAGTTC	165
Egr1	AGGTGACACTATAGAATAGGTGGAGACGAGTTATCCCA<br>GTACGACTCACTATAGGGAAGGCCACTGACTAGGCTGAA	172
Nr5a1	AGGTGACACTATAGAATACCCCAGAGGATACCATGAGA<br>GTACGACTCACTATAGGGAGATAAATACCAGCCCAGCCA	242
Nr0b1	AGGTGACACTATAGAATATCCTGTACCGCAGCTATGTG<br>GTACGACTCACTATAGGGACCACCTGTGGATCCTTGAGT	214
β-Actin	AGGTGACACTATAGAATAGTACCACCATGTACCCAGGC<br>GTACGACTCACTATAGGGAGTACTTGCGCTCAGGAGGAG	144
Gnrhr	AGGTGACACTATAGAATACTTGATACAGGGCAAGCTCC<br>GTACGACTCACTATAGGGAGCACCTTCATCCTTGAGAGC	207
KanR	AGGTGACACTATAGAATAATCATCAGCATTGCATTCGATTCCTGTTTG<br>GTACGACTCACTATAGGGAATTCCGACTCGTCCAACATC	325

Supplemental Table S1: Primer sequences for multiplex RT-qPCR assays for natriuretic peptide genes, and gonadotrope transcription factor genes. Murine mRNA sequences were obtained from NCBI Nucleotide (https://www.ncbi.nlm.nih.gov/nuccore), were imported into express Designer Software (Beckman Coulter).

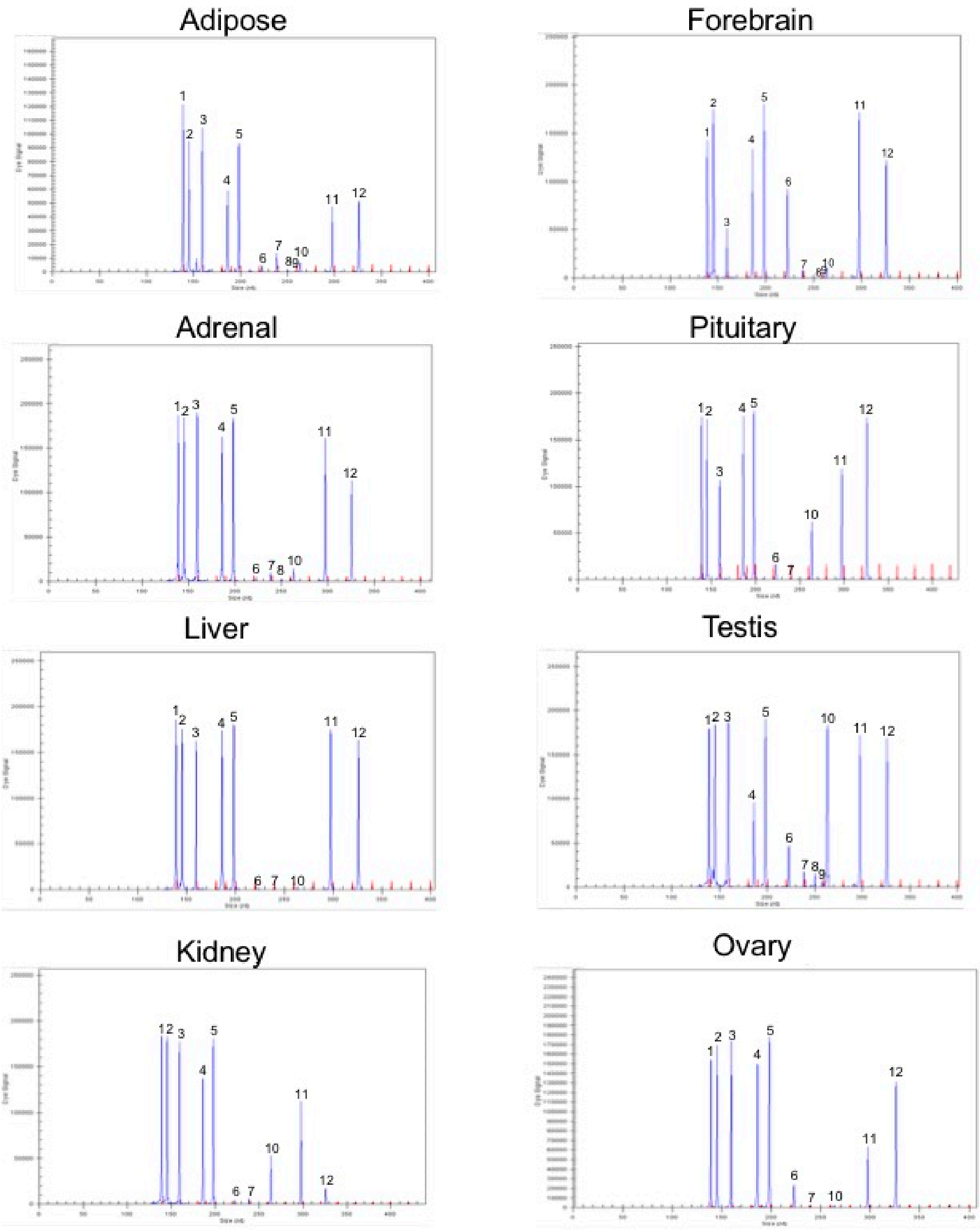

Supplemental Figure S1: Representative electropherograms of multiplex RT-qPCR analyses of expression for natriuretic peptides (*Nppa, Nppb, Nppc*), natriuretic peptide receptors (*Npr1, Npr2, Npr3*), and proconvertase enzymes (*Furin* and *Corin*) (blue peaks). Total RNA was extracted from adipose, adrenal, liver, kidney, forebrain, pituitary, testis and ovary. Genes were detected in order of size corresponding to size standards run alongside the products (140nt-420nt) (red peaks) and quantified by capillary electrophoresis.

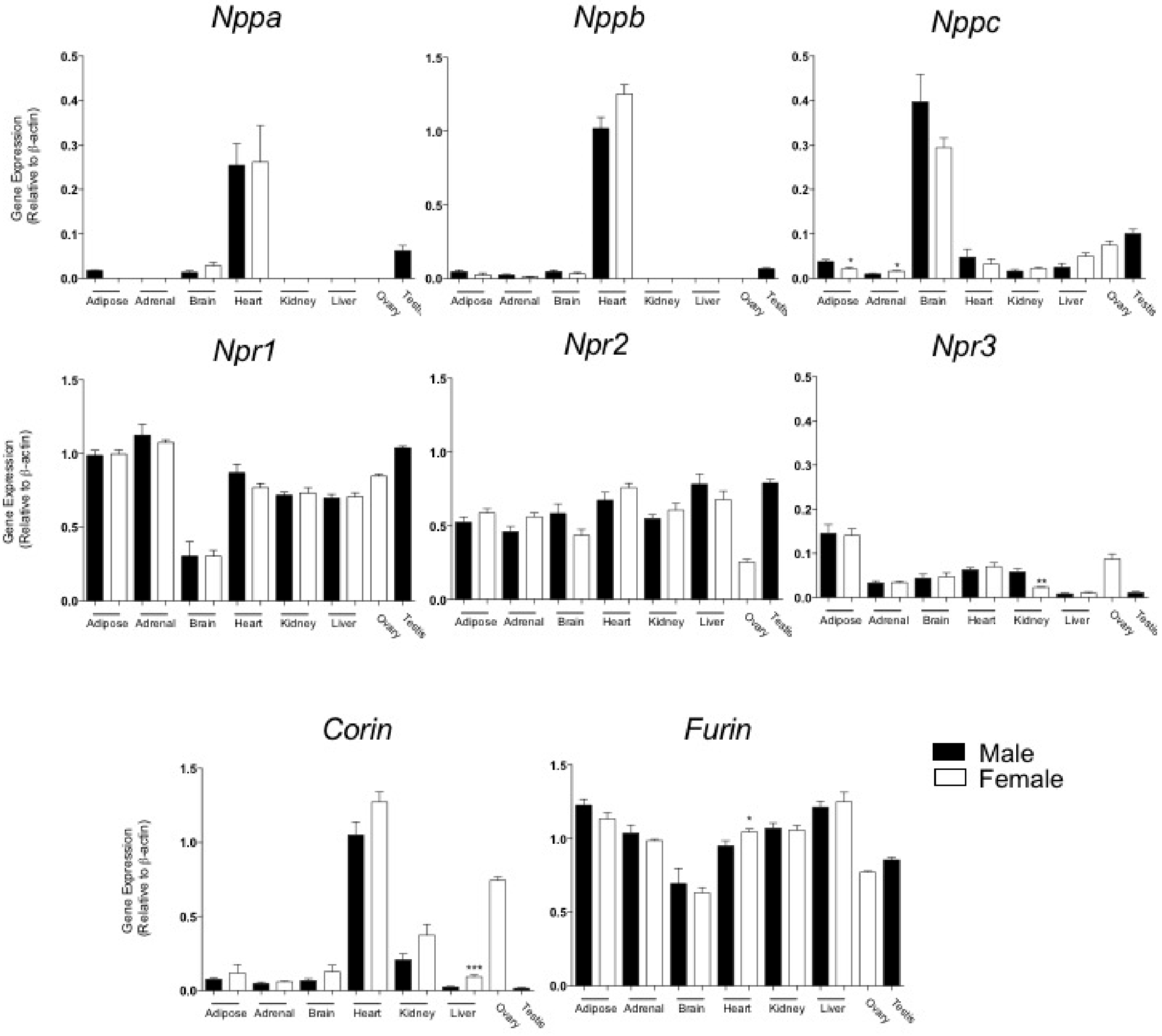

Supplemental Figure S2: Multiplex RT-qPCR data of natriuretic peptide gene expression from murine adipose, adrenal, liver, kidney, forebrain, pituitary, testis and ovary. Tissue was collected from n=5 to n=8, 12 week-old C57/B6 males or females and total RNA extracted.

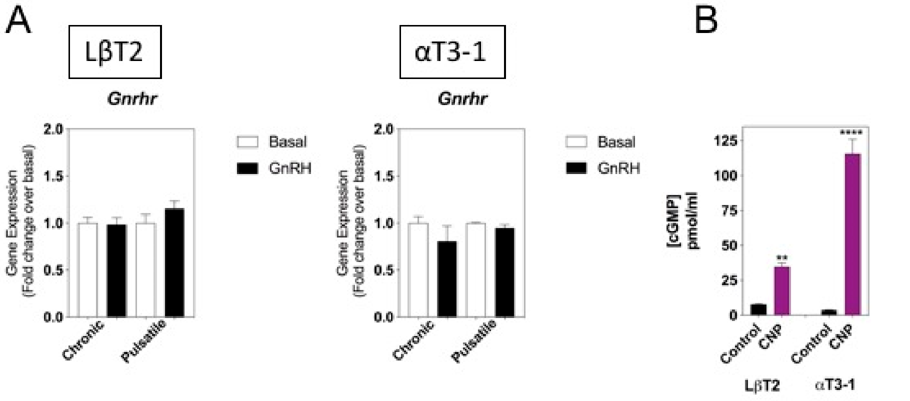

Supplemental Figure S3 A) Effect of continuous or pulsatile GnRH stimulation on *Gnrhr* expression in LβT2 and αT3-1 cell lines. Cells were treated with 0 or 100nM GnRH, for either 4hr continuously, or as 5 min pulses every hour for 4hr, before extracting RNA and performing multiplex RT-qPCR for *Gnrhr* (as part of the same multiplex described in Figure 4). Data shown are means±SEM (n= 6 to 9 individual RNA extractions) of relative gene expression (normalized to

ActB). **B**) Total cGMP accumulation in LβT2 and αT3-1 cells treated with 0 or 100nM CNP for 1h in physiological saline solution containing 1mM IBMX, before measuring with a commercially available cGMP-EIA kit (R&D Systems) as described previously [8]. Data shown are means±SEM representative of three independent experiments, each performed in triplicate. **P<0.01, ****P<0.0001, significantly different from Control cells.
